# Assessment of anterior-posterior spinal curvatures in children suffering from hypopituitarism

**DOI:** 10.1186/s12902-019-0462-4

**Published:** 2019-12-11

**Authors:** Magdalena Kobylińska, Roksana Malak, Katarzyna Majewska, Andrzej Kędzia, Włodzimierz Samborski

**Affiliations:** 1 Department of Clinical Auxology and Pediatrics Nursing Faculty of Health Sciences, Poznań University of Medical Sciences, Szpitalna 27/33, 60-572 Poznań, Poland; 2 Department and Clinic of Rheumatology and Rehabilitation, Poznan University of Medical Sciences, 28 Czerwca 1956 nr 135/147, 61-545 Poznań, Poland

**Keywords:** Spinal curvatures, Body posture, Inclinometer

## Abstract

**Background:**

Body posture may be disordered by vestibular dysfunction, neurological disorders, problems with the distribution of muscle tone, brain injuries, and other dysfunctions. Growth hormone deficiency (GHD) can lead to many disorders, particularly of the musculoskeletal system. During treatment with recombinant human growth hormone (rhGH), an increase in muscle mass and an improvement in bone structure can be observed in children suffering from hypopituitarism from GHD.

**Methods:**

The study involved 33 children suffering from hypopituitarism with GHD (9 girls and 24 boys), aged 10–14 years old. Measurements of the magnitude of their anterior–posterior spinal curvatures were made using an inclinometer. The children were examined at the medianus of the sacrum bone, the Th12–L1 intervertebral area, and the C7–Th1 intervertebral area. In order to characterize the anterior–posterior curvature of the spine, the results were compared with the general norms reported by Saunders. Statistical calculations were carried out using the statistical package Statistica 10 PL.

**Results:**

Lumbar lordosis angles were higher in the patients currently receiving growth hormone (GH) treatment than in those who had yet to receive it. There is a statistically significant positive correlation between the length of growth hormone treatment and the alpha angle. There are also statistically significant correlations between age at the beginning of growth hormone therapy and the angle of lordosis. Statistically significant correlations were also seen between age at the beginning of growth hormone therapy and the alpha angle.

**Conclusions:**

Although there may be changes in posture at the beginning of rhGH treatment, the sooner growth hormone therapy begins, the better the body posture. The longer the growth hormone treatment, the better the posture, as expressed by the alpha angle in the sagittal plane.

## Background

There is no one universal definition of body posture. The mechanisms that account for it include postural tone, reciprocal innervation, righting, and equilibrium reaction [1]. From the biomechanical point of view, body posture can be described as the mechanical capacity for kinesthetic sense and muscle balance [1]. For this reason, it is interesting to determine what the body posture looks like when the biomechanical parameters are less typical, as in the case of children who are shorter and whose proportions may be different because of growth hormone deficiency (GHD).

Postural mechanisms are responsible not only for the position of the body, but also for the location of each postural segment, particularly the physiological curvature of the spine, the position of the head and the cervical spine, and the position of the pelvis [1]. Maintaining body posture alignment, by maintaining appropriate relationships between body segments so as to counteract gravity, should in turn lead to maintenance of the upright position [2]. If we regard posture in this way, we can monitor body posture in children with a range of disorders, such as cerebral palsy and GHD. However, there are no extant studies of body posture in children with GHD.

Disorders of posture may cause dysfunctions that affect quality of life [3]. Certain severe posture dysfunctions can affect the basic vital functions, such as breathing, swallowing, and maintaining the body against gravity [4]. Maintenance of correct body posture requires the proper distribution of muscle tone. The antigravity muscles in particular play important roles in maintaining vertical position. They also enable the continuous recovery of balance during motor activities [5]. Postural control is very important during daily activities, because it allows muscles to operate ergonomically in position, which is comfortable and effortless [5]. Body posture may be disordered by vestibular dysfunction, neurological disorders, muscle contractures, brain injuries, or other dysfunctions. A range of pathological mechanisms can provoke postural disorders [6]. Even environmental and behavioral aspects, such as a reduction of physical activity, may lead to postural disorders, especially in children and adolescents [7].

Physical development is important for the efficient functioning of *human beings*. Short stature can unfavorably distinguish a child from his or her peers, disturbing psychosocial development and thus affecting participation in society. Many children with short stature have no other features of disease; they only demonstrate a delay in the processes of growth and maturation [8, 9]. However, when this is caused by GHD, many other associated disorders besides short stature may occur, especially of the musculoskeletal system. GHD in childhood is associated with altered body composition, leading to increased body fat mass and a decrease in lean body mass, which is an essential component of muscle mass. Children with GHD also often present differently from typically developing children, showing bone architecture with low bone mineral density parameters and bone mineral contents. Treatment with recombinant human growth hormone (rhGH) can lead to an increase in muscle mass and improvements in bone structure. GHD also seems to be associated with poor motor skills [10–12].

The aim of this study was to assess anterior–posterior spine curvature in short-statured children diagnosed with hypopituitarism and GHD, and to determine whether therapy with recombinant human growth hormone may be a risk factor for postural defects in children.

## Methods

The research received the consent of the Bioethical Commission at Poznań University of Medical Science (no. 1107/17, 9 November 2017).

### Characteristics of the research group

The study involved 33 children with short stature, all patients from Karol Jonscher Clinical Hospital in Poznań. The research group consisted of 9 girls and 24 boys, aged 10–14. The study group included six children who had been just admitted for growth hormone treatment and 27 patients undergoing long-term growth hormone treatment, with different durations of therapy (1 to 8 years). Data describing the group are presented in Table 1. Body posture was assessed by the same person, a qualified physiotherapist experienced in working with children with posture failure, and who had received specialist education.

**Table 1:** Study group characteristics

	Girls	Boys	Total
Number of children	9 (27.3%)	24 (72.7%)	33
Body mass [kg]	30.9 ± 9.9	35.7 ± 11.9	34.4 ± 11.4
Height [cm]	134.5 ± 10.1	139.8 ± 12.7	138.3 ± 12.1
BMI [kg m^2^]	16.74 ± 3.10	17.85 ± 3.04	17.55 ± 3.05
Age at start of treatment [years]	8.11 ± 2.10	8.10 ± 2.02	8.11 ± 2.01
Treatment length [years]	1.97 ± 1.96	3.23 ± 2.05	2.89 ± 2.08
Sport [h]	3.56 ± 3.50	4.09 ± 4.65	3.94 ± 4.32
Screen time [h]	1.83 ± 0.61	2.21 ± 1.38	2.11 ± 1.22

The inclusion criterion was a diagnosis of GH deficiency (somatotropin pituitary insufficiency). Patients with neurological deficits, genetic defects, or orthopedic disease, or who had undergone invasive surgical procedures, were excluded, as were patients with traumatic perinatal history, encephalopathy, tumor, or traumatic brain injury.

Table 1 presents the characteristics of the group, including body weight, height, body mass index (BMI), age at recombinant growth hormone treatment, duration of the treatment, number of hours per week spent on sports, and number of hours daily spent watching television or using mobile phones.

### Assessment procedure

Firstly, the parent or caregiver was asked to fill in a questionnaire providing personal data, including a perinatal interview, information on the amount of time the child spends on sports activities and the number of hours spent watching television or using mobile phones. In addition, the questionnaire asked about the current state of health of the child, including whether he or she has any chronic diseases or vision or hearing deficiency or has undergone surgery. The questionnaire was developed for the purpose of this study.

Weight and height were measured using a medical height meter with a weight (Radwag 2006). Height was measured to an accuracy of 0.1 cm and weight to 0.01 kg.

The spine was assessed in the sagittal plane in a free standing position, without shoes. The lower limbs were straight at the knee joints, and the feet were placed the width of the hips apart. The upper limbs rested comfortably along the body. The subject was asked to look at one point, located at eye level.

In all patients, the spinal processes of the vertebrae were marked with a nontoxic marker. A baseline mechanical inclinometer was used to measure the anterior–posterior curvature of the spine [13–16].

Three angles were measured:

Alpha: inclination of the lumbosacral segment; the center of the inclinometer was placed at the midpoint of the sacrum.

Beta: slope of the thoracolumbar segment; the center of the inclinometer was placed on at the Th12–L1 intervertebral space.

Gamma: slope of the upper thoracic segment; the center of the inclinometer was placed at the C7–Th1 intervertebral space [17].

The lumbar lordosis angle was determined by adding Alpha and Beta.

Thoracic kyphosis was taken as the sum of Beta and Gamma [13].

To describe the anterior–posterior curvature of the spine, the results were compared with the general norms provided by Saunders (Table 2) [18].

**Table 2:** Saunders’s norms [18]

Measurement	Norms
Sacroiliac angle [^o^]	15–30
Lumbar lordosis angle [^o^]	30–40
Thoracic kyphosis angle [^o^]	30–40

The parametric Student’s *t*-test was used to check the difference between boys and girls for the angle of lumbar lordosis and the alpha angle. The Mann–Whitney *U*-test was used to determine the difference in the thoracic angle. Correlations between variables were determined by the significance test of Spearman’s rank correlation coefficient. A *p*-value < 0.05 was considered statistically significant. Statistical calculations were carried out using the statistical package Statistica 10 PL.

## Results

Table 3 presents the number and percentage of children who presented the norm or a deviation from the norm—that is, an increased or decreased alpha angle (angle of the sacrum), in comparison to Saunders’s standards (Table 2).

**Table 3:** The sacrum angle compared to Saunder’s norms

	*n*	%
Increased angle > 30	1	3
Norm 15–30	27	82
Decreased angle < 15	5	15

Table 4 presents the number and percentage of children who embodied the norm or showed a deviation from the norm, in the form of increased or decreased angles of kyphosis and lumbar lordosis, compared to Saunders’s standards (Table 2).

**Table 4:** Kyphotic and lumbar lordosis angles compared to Saunder’s norms

	Kyphotic angle		Lumbar lordosis angle	
	*n*	%	*N*	%
Increased angle > 40	10	30	6	18
Norm 30–40	22	67	19	58
Decreased angle < 30	1	3	8	24

Table 5 shows the average value of alpha angle, thoracic kyphosis angle, and lumbar lordosis in research group.

**Table 5:** The average value of alpha angle, thoracic kyphosis angle, and lumbar lordosis

Parameter	*n*	Mean value	Standard Deviation	Median	Min.	Max.
Kyphotic angle [°]	33	39.5	8.4	38.0	28	55
Lumbar lordosis angle [°]	33	32.8	9.1	32.0	10	50
Alpha angle [°]	33	19.8	6.9	20.0	5	35

There were no statistically significant differences between boys and girls in the angle of thorax kyphosis, lumbar lordosis and alpha angle (statistical significance where considered if *p* > 0.05) (Table 6).

**Table 6:** The mean value of alpha angle, thoracic kyphosis angle and lumbar lordosis angle in boys and girls and results of tests of significance of differences between them

Parameter	Gender	*n*	Mean Value	SD	Median	Min.	Max.	U / t (df)	*p*
Kyphotic angle [°]	girls	9	38.1	10.2	35.0	28	55	83.5	0.3320^(a)^
	boys	24	40.1	7.7	39.0	30	55		
Lumbar lordosis angle [°]	girls	9	31.0	11.8	32.0	10	50	−0.71 (31)	0.4818^(b)^
	boys	24	33.5	8.0	32.5	15	45		
Alpha angle [°]	girls	9	18.3	8.7	20.0	5	35	−0.72 (31)	0.4778^(b)^
	boys	24	20.3	6.3	20.0	10	30		

^(a)^: Mann–Whitney *U*-test results

^(b)^: Student’s *t*-test results

U: Mann–Whitney *U*

t: Student’s *t*

df: degrees of freedom in Student’s *t-*test

p: probability

**Fig. 1:**
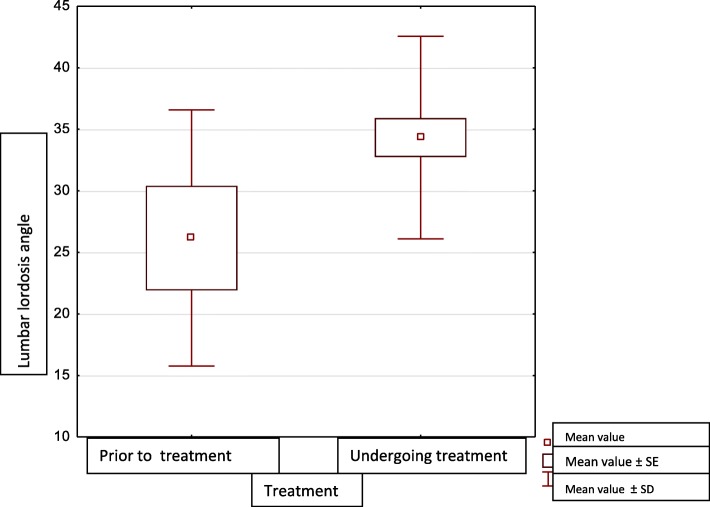
Lumbar lordosis angle in patients who had not yet received growth hormone treatment and those who were receiving it

There was a statistically significant difference in the lumbar lordosis angle between patients prior to treatment and patients undergoing treatment (*p* = 0.0439), (Fig. 1). Higher lumbar lordosis angles were found in patients undergoing growth hormone treatment than in those who had not yet undergone it (Table 7).

**Table 7:** Mean values ​​of thoracic kyphosis, lumbar lordosis, and alpha angles in patients prior to and during growth hormone treatment and the results of tests of significance of differences between them

Parameter	Treatment	*n*	Mean Value	SD	Median	Min.	Max.	U / t (df)	*p*
Kyphotic angle [°]	Before treatment	6	38.0	10.2	35.0	28	55	64.0	0.4412^(a)^
	During treatment	27	39.9	8.1	38.0	30	55		
Lumbar lordosis angle [°]	Before treatment	6	26.2	10.4	27.5	10	40	−2.10 (31)	0.0439*^(b)^
	During treatment	27	34.3	8.2	35.0	15	50		
Alpha angle [°]	Before the treatment	6	16.3	8.2	17.5	5	28	−1.36 (31)	0.1844^(b)^
	During treatment	27	20.5	6.5	20.0	10	35		

^(a)^: Mann–Whitney *U*-test results

^(b)^: Student’s *t*-test results

U: Mann–Whitney *U*

t: Student’s *t*

df: degrees of freedom in Student’s *t-*test

p: probability

*: statistically significant, *p* < 0.05

There were no statistically significant differences in the thoracic kyphosis and alpha angles between patients who had not undergone treatment and those receiving treatment (*p* > 0.05).

There were no statistically significant differences between body weight, height, and BMI index and thoracic kyphosis, lumbar lordosis, and alpha angles (p > 0.05). The results are presented in Table 8.

**Table 8:** The results of the significance test of Spearman’s rank correlation coefficient between body mass, height, and BMI index and thoracic kyphosis, lumbar lordosis, and alpha angles

Parameters	*n*	Rs	t(*n*-2)	*p*
Weight [kg] & kyphotic angle [°]	33	0.251	1.44	0.1589
Weight [kg] & lumbar lordosis angle [°]	33	0.048	0.27	0.7901
Weight [kg] & alpha angle [°]	33	0.143	0.80	0.4288
Height [cm] & kyphotic angle [°]	33	0.281	1.63	0.1128
Height [cm] & lumbar lordosis angle [°]	33	0.020	0.11	0.9141
Height [cm] & alpha angle [°]	33	0.035	0.19	0.8474
BMI [kg/m^2^] & kyphotic angle [°]	33	0.115	0.65	0.5224
BMI [kg/m^2^] & lumbar lordosis angle [°]	33	−0.065	−0.36	0.7207
BMI [kg/m^2^] & alpha angle [°]	33	0.200	1.14	0.2647

Rs: Spearman’s rank correlation coefficient for the number n

t: *t*-statistic for the significance of the Rs coefficient for n-2 degrees of freedom

p: probability

The relationship between the number of hours per week spent in sport activities and the number of hours daily spent in watching TV and using mobile devices, and the thoracic kyphosis, lumbar lordosis, and alpha angles were also examined. There were no statistically significant correlations (p > 0.05) between these variables.

There was a statistically significant positive correlation between the length of growth hormone treatment and the alpha angle. The longer the duration of therapy, the greater the alpha angle.

We found no significant correlation between the angle of thoracic kyphosis or the angle of lumbar lordosis and the duration of growth hormone treatment (Table 9).

**Table 9:** Results of significance test of Spearman’s rank correlation coefficient between length of growth hormone treatment and thorax kyphosis, lumbar lordosis, and alpha angles

Correlation between two parameters	*n*	Rs	t(*n*-2)	*p*
Length of growth hormone treatment [years] & kyphotic angle [°]	33	0.144	0.81	0.4224
Length of growth hormone treatment [years] & lumbar lordosis angle [°]	33	0.308	1.80	0.0817
Length of growth hormone treatment [years] & alpha angle [°]	33	0.362	2.16	0.0382*

Rs: Spearman’s rank correlation coefficient for the number n

t: *t*-statistic for the significance of the Rs coefficient for n-2 degrees of freedom

*p*: probability

*: statistically significant, *p* < 0.05

There was a statistically significant negative correlation between age at the beginning of growth hormone therapy and angle of lordosis. The sooner children began therapy, the smaller the angle of lordosis. A statistically significant negative correlation was also found between age at the beginning of growth hormone therapy and the alpha angle, which means that the earlier therapy began, the lower the alpha angle. No significant correlation was found between angle of thoracic kyphosis and the age of initiation of therapy (Table 10).

**Table 10:** The results of the Spearman’s rank correlation coefficient test between age at the beginning of therapy and angle of thorax kyphosis, angle of lumbar lordosis, and alpha angle

Correlation between two parameters	*n*	Rs	t(*n*-2)	*p*
Age at beginning of growth hormone treatment [years] & kyphotic angle [°]	33	0.007	0.04	0.9685
Age at beginning of growth hormone treatment [years] & lumbar lordosis angle [°]	33	−0.350	−2.08	0.0462*
Age at beginning of growth hormone treatment [years] & alpha angle [°]	33	−0.460	−2.89	0.0070*

Rs: Spearman’s rank correlation coefficient for the number n

t: *t*-statistic for the significance of the Rs coefficient for n-2 degrees of freedom

p: probability

*: statistically significant, p < 0.05

## Discussion

Higher values of the lumbar lordosis angle were found among the patients undergoing growth hormone treatment than in among patients who had not yet undergone it. There was a statistically significant correlation between the duration of growth hormone treatment and the alpha angle. There were statistically significant correlations between age at the beginning of growth hormone therapy and lordosis angle. Statistically significant correlations were also seen between age at the beginning of growth hormone therapy and the alpha angle.

Therapy with human recombinant growth hormone has been available since the 1980s. There is no doubt that this treatment improves the condition of patients, and can even reverse most signs and symptoms of this hormonal deficiency. However, chronic GH administration may be associated with risks of increasing BMI, waist circumference, waist–hip ration, and even cardiovascular risk markers [19].

There have been studies that have shown progression of scoliosis under GH treatment [20–22]. However, there has been no research into how GH might affect spinal curvature in the sagittal plane. Czaprowski et al. investigated the curvature of the anterior–posterior spine using a Saunders digital inclinometer in 249 healthy children aged 10–14 years. They obtained average thoracic kyphosis and lumbar lordosis values ​​of 42.6° and 34.5° in females, and 42.9° and 31.2° in boys. The mean inclination angle of the sacrum was 19.3° [23]. That research also showed a possible problem in ​​lumbar lordosis formation and the angle of pelvis tilt among patients. Patients undergoing treatment with recombinant growth hormone had greater lumbar lordosis angles than did patients who had not yet undergone treatment. Increased pelvic anteversion may be associated with a weakness of the abdominal muscles. Under typical conditions, correct contraction of abdominal muscles should lead to the front part of the pelvis being lifter [24]. Furthermore, patients with GH deficiency may accumulate fat in a central (abdominal) distribution, which could be associated with hyperlordosis in the lumbar spine [25]. It could affect not only posture, but also general wellbeing and, in particular general fitness.

Stupnicki conducted the Eurofit fitness test on girls with Turner syndrome, in whom symptoms of GHD are common. The subjects had weaker results in strength tests than did healthy peers. They also had worst results in the lying-down test, which indicates weak abdominal muscles in these girls [26]. These girls with GHD symptoms showed problems changing body position from horizontal to sitting, which engages the abdominal muscles. Physical fitness and individual motor skills have not yet been studied among children with hypopituitarism.

We have shown a relationship between age at the beginning of GH therapy and body posture in the sagittal plane. Patients who began treatment with growth hormone earlier presented a smaller angle of lumbar lordosis and smaller sacral angle than those who began GH therapy later.

There is a great need for further research on patients with GHD. However, we believe that our results will prove useful in the everyday practice of clinicians who look after of children with hypopituitarism. It seems reasonable that patients currently undergoing therapy with recombinant growth hormone should be under a physiotherapist’s care, in order to monitor posture, to allow early intervention when abnormalities are detected, and to recommend appropriate preventative measures.

Future studies are needed on the body posture of patients with GHD. It is clear that other factors affect the body posture, including muscle strength. For this reason, more studies should be carried out on other important issues, such as muscle strength. Furthermore, scoliosis and physical activity would be interesting subjects of further studies.

## Conclusions

Although there may be some changes in posture in the beginning of the growth hormone therapy, the sooner the growth hormone therapy begins the better the resulting body posture.

The longer the growth hormone treatment, the better the posture in terms to the alpha angle in the sagittal plane.

Our next study will involve measuring scoliosis, muscle strength, and physical activity.

## Data Availability

The datasets used and analysed during the current study are available from the corresponding author on reasonable request.

## Abbreviations

BMI: Body Mass Index
C7: Seventh cervical vertebra
cm: Centimetre
df: Degees of freedom for t-Student test
GHD: Growth hormone deficiency
h: Hours
kg: Kilogram
L1: First lumbar vertebra
m: Metre
max: Maximum
min: Minimum
n: Number
p: Probability
rhGH: Recombinant human growth hormone
Rs: Spearman’s rank correlation coefficient for the number
SD: Standard deviation
SE: Standard error
t: Statistic value of t-Student test
Th1: First thoracic vertebra
Th12: Twelfth thoracic vertebra
TV: Television
U: Statistic analyzis of U Mann-Whitney test
